# Effect of antihypertensive medication reduction on short-term blood pressure control in older adults: calibration of OPTiMISE trial results to real-world primary care data

**DOI:** 10.1093/ageing/afag192

**Published:** 2026-07-01

**Authors:** Antoine Christiaens, Ariel Wang, David A McAllister, Richard J McManus, James P Sheppard

**Affiliations:** Nuffield Department of Primary Care Health Sciences, University of Oxford, Oxford, UK; Louvain Drug Research Institute/Clinical Pharmacy and Pharmacoepidemiology Research Group, Université catholique de Louvain, Brussels, Belgium; Nuffield Department of Primary Care Health Sciences, University of Oxford, Oxford, UK; School of Health and Wellbeing, University of Glasgow, Glasgow, UK; Brighton and Sussex Medical School, University of Sussex, Brighton, UK; Nuffield Department of Primary Care Health Sciences, University of Oxford, Oxford, UK

**Keywords:** older adults, antihypertensive treatment, deprescribing, calibration, statistical transportability

## Abstract

**Background:**

While antihypertensive treatment prevents cardiovascular events, it may also increase risks such as falls in patients with frailty. The Optimising Treatment for Mild Systolic Hypertension in the Elderly (OPTiMISE) trial found that deprescribing one antihypertensive drug did not result in worse short-term blood pressure control and long-term follow-up showed no observed harm, but its generalisability to routine clinical practice remains uncertain.

**Objective:**

This study aimed to calibrate the OPTiMISE effect to a representative primary care population in England.

**Methods:**

We calibrated the OPTiMISE treatment effect using inverse probability weighting (IPW) based on trial inclusion likelihood. The trial enrolled 569 adults aged ≥80 years with controlled blood pressure on ≥2 antihypertensive drugs. A target population was reconstructed from electronic health records of 24 participating practices and extrapolated to all English adults aged ≥80 years, prescribed ≥2 antihypertensive drugs, using NHS Digital data. The primary outcome was all-cause hospitalisation or death. Weighted Cox models estimated calibrated hazard ratios (HRs).

**Results:**

The target population included 798 179 individuals (median age 84 years [81–87], 48% female). Compared with OPTiMISE participants, a higher proportion of individuals in target population were overtly frail (25% vs. 11%). After calibration using IPW, deprescribing was not associated with higher risk of hospitalisation or death [calibrated HR 0.94 (95% CI: 0.73–1.22)], similar to the long-term follow-up of OPTiMISE [HR 0.93 (95% CI: 0.76–1.12)], although with a slightly wider confidence interval.

**Conclusions:**

Calibrating OPTiMISE findings to a representative primary care population frailer than the original participants suggests that the original trial findings could be translated into a real-world population.

## Key Points

- The real-world target population had great levels of frailty and more co-morbid conditions than OPTiMISE trial participants.
- After calibration using inverse probability weighting, deprescribing one antihypertensive was not associated with increased risk of hospitalisation or death.
- Findings support the transportability of OPTiMISE long-term results to a community-dwelling population of very old adults.

## Introduction

Hypertension is one of the most prevalent chronic conditions in older people, affecting more than half of those aged 80 years or more [1, 2]. While the benefits of antihypertensive treatment are well established in younger and healthier patients (e.g. in preventing stroke and cardiovascular disease), those might be outweighed by risks of treatment-related complications in older adults (e.g. falls with fractures, hospitalisations or death), particularly among the individuals living with frailty and long-term conditions [3–5].

To address this uncertainty, the Optimising Treatment for Mild Systolic Hypertension in the Elderly (OPTiMISE) trial randomised people aged 80 and older taking two or more antihypertensives to deprescribing versus usual care [6, 7]. During long-term follow-up following the initial short-term primary outcome of blood pressure control, there was no evidence of an increased risk of hospitalisation or death in the deprescribing arm, despite half of patients still taking fewer antihypertensive medications 4 years after randomisation [7]. However, inclusion in the OPTiMISE trial, as any other trial, might be subject to selection bias, potentially leading to low representativity of the general population (i.e. lack of external validity) [8–10]. In particular, vulnerable older people, those living with frailty and multiple-long term conditions, were likely to be underrepresented in this trial [6, 11].

To address this problem, we proposed to statistically ‘calibrate’ OPTiMISE’s findings by re-weighting the trial population to reflect the characteristics of a representative community-dwelling primary care population [12]. This approach, while not breaking randomisation, provides a single trial effect estimate which accounts for differences between trial participants and the wider population.

## Methods

### Study design

**Figure 1:**
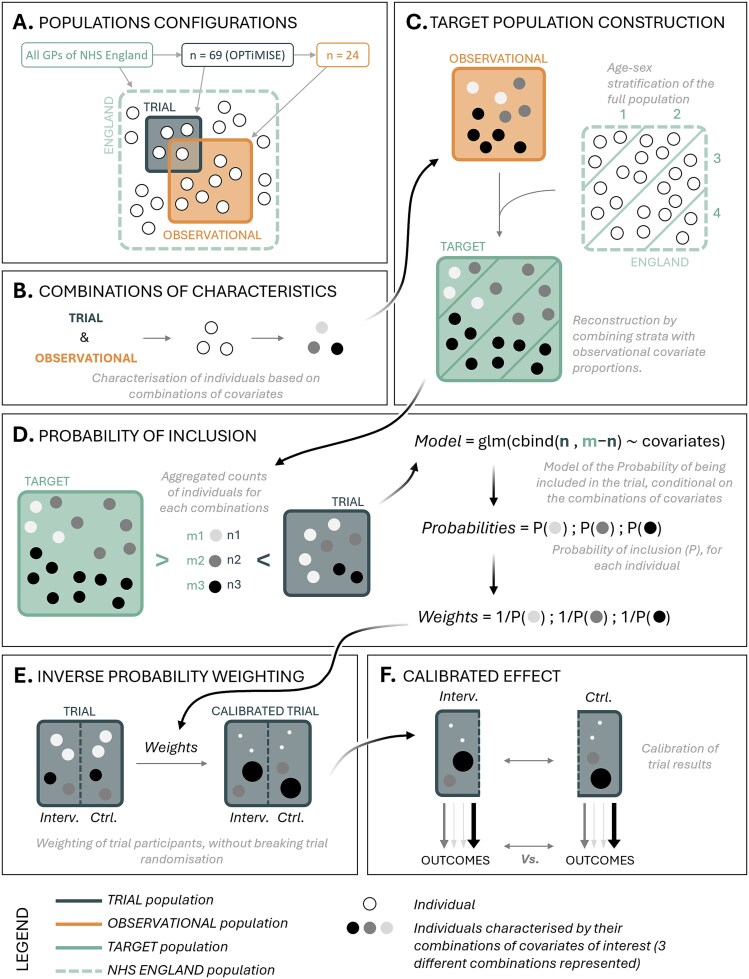
Framework for calibrating OPTiMISE trial results using real-world data. GP, general practice; NHS, National Health Service; Interv., intervention group; Ctrl., control group.

This study was designed as a trial calibration (or transportability) study [13–15] to generalise the treatment effect observed in the randomised OPTiMISE trial [7] to a real-world target population derived from observational routine primary care data [11] (Figure 1).

### Study populations

The trial population comprised participants enrolled in the OPTiMISE trial, conducted across 69 primary care centres in England. Eligible participants were aged 80 years or older, had a clinical systolic blood pressure (SBP) <150 mmHg, and were treated with ≥2 antihypertensive medications (i.e. ACE inhibitors, angiotensin II receptor blockers, calcium channel blockers, thiazide or thiazide-like diuretics, potassium-sparing diuretics, alpha-blockers or beta-blockers) for at least 12 months before baseline with a stable dosage for ≥4 weeks. Participants provided informed consent and, in the investigator’s opinion, were able to comply with the trial requirements and likely to benefit from antihypertensive reduction. Participants were randomised (1:1) to antihypertensive medication reduction (i.e. removal of one antihypertensive) or usual care.

The real-world target population consisted of patients aged 80 years or older from the 24 of the 69 primary care centres participating in the OPTiMISE trial that used Egton Medical Information Systems (EMIS) search and reporting software (Egton Medical Information Systems Health, Leeds, UK) (Figure 1A). The patients were originally included in a cross-sectional study, with data extracted from routine electronic health records between September 2017 and October 2018 [11]. We restricted inclusion to patients currently treated with ≥2 antihypertensive medications daily to ensure greater alignment with trial participants.

The age-sex distribution of the English primary care population (of whom both the observational data and trial data are a subset) was obtained using publicly available data from NHS Digital [2].

### Covariates

Baseline covariates were selected based on their potential influence on trial participation and treatment effects: age, systolic and diastolic blood pressure (SBP and DBP), body mass index, frailty category (fit, less fit and frail), cardiovascular polypharmacy (≥3 cardiovascular medications/day), chronic kidney disease, type 2 diabetes, previous myocardial infarction and history of stroke or transient ischaemic attack [7, 11]. All covariates were harmonised and categorised into predefined groups to ensure consistency between datasets. In particular, the definition of frailty was harmonised across datasets by mapping the electronic frailty index (trial) and the claims-based frailty score (observational cohort) to a common set of frailty categories (fit, less fit and frail) [11].

### Outcomes

Outcomes were assessed exclusively within the trial population and corresponded to those pre-specified in the original OPTiMISE trial [7]. The primary outcome was a composite of all-cause hospitalisation or death from any cause. Secondary outcomes included all-cause mortality, first all-cause hospitalisation, emergency hospitalisation and hospitalisation due to major cardiovascular events, myocardial infarction or stroke. Although the trial included an active follow-up period of 3 months after randomisation, participants were subsequently followed passively through linkage to electronic health records for a mean duration of 4.1 years, with outcomes ascertained using Hospital Episode Statistics and civil registration death data from NHS England. All outcomes were evaluated over the 4-year period following randomisation.

### Statistical analysis

#### Estimation of trial inclusion probabilities

Trial and observational populations were each aggregated across combinations of covariates of interest, enabling the count of individuals sharing identical combinations within each dataset (Figure 1B). To implement inverse probability weighting (IPW) for trial calibration, all covariate combinations observed in the target population ideally need to be represented in the trial population to ensure stable estimation of inclusion probabilities [16]. Combinations absent in the target population were therefore excluded from model estimation to avoid violations of the positivity assumption [17].

The trial population was neither completely separate from nor a subset of the observational population (some but not all trial participants appeared in both; Figure 1A). Hence, we used the observational population data plus the publicly available aggregate-level data to construct a target population representing the full target population at the scale of England. Within each age-sex stratum, we applied the proportions of clinical and treatment characteristics from the observational data, assuming representativeness conditional on age and sex (Figure 1C). This approach yielded stratum-specific estimates of the number of individuals for each covariate combination in the target population. These reconstructed counts were subsequently aggregated across all strata to form the virtual target population. Thus, the trial populations were a subset of the target population, allowing us to estimate inclusion probabilities.

#### Probability of inclusion and IPW

The probability of inclusion in the trial was estimated using a logistic regression model fitted on the aggregated trial and target data, describing the likelihood that an individual with specific baseline characteristics belonged to the trial population (Figure 1D). For each participant, we obtained a model-derived predicted probability of inclusion based on a logit transformed linear combination of covariates and their estimated coefficients [14, 18].

IPWs were then derived as the inverse of these predicted probabilities, assigning greater weight to underrepresented profiles and smaller weight to overrepresented ones. To improve model stability, stabilised IPWs were obtained by multiplying each weight by the marginal probability of trial participation in the combined dataset. Predicted probabilities below 1% were truncated to prevent extreme values from unduly influencing estimates [14]. The resulting stabilised weights were applied to the trial data to reweight its covariate distribution towards that of the virtual target population (Figure 1E).

#### Calibration of trial effects using IPW

Treatment effects from the OPTiMISE trial were re-estimated using stabilised IPWs (Figure 1F). Weighted analyses were conducted using Cox proportional hazards models adjusted for baseline SBP, with robust standard errors to allow for clustering of individuals within practices. Estimates were reported as hazard ratios (HRs) with corresponding 95% confidence intervals.

#### Sensitivity analyses

A sensitivity analysis was conducted to evaluate the robustness of the calibration results to the choice of covariates used in the construction of the inclusion model. Specifically, frailty was removed from the set of baseline characteristics included in the probability-of-inclusion models.

#### Statistical software and reproducibility

All analyses were conducted using R (version 4.5.1) [19]. Aggregation of covariate combinations, estimation of inclusion probabilities, construction of stabilised IPWs, and weighted Cox regression models were implemented using base R and dedicated packages. All summary statistics used in the calibration procedure (i.e. aggregated counts), the fitted model outputs (regression coefficients and variance–covariance matrices), and the full analysis code are openly available at GitHub [20].

### Ethics

The study was approved by a National Health Service (NHS) Research Ethics Committee (South Central—Oxford A; 16/SC/0628) and the Medicines and Healthcare products Regulatory Agency (MHRA; 21584/0371/001-0001). All participants gave written informed consent. This study is registered with the International Standard Randomised Controlled Trial Number registry (ISRCTN97503221) and the European Union Drug Regulating Authorities Clinical Trials Database (EudraCT2016-004236-38).

## Results

**Figure 2:**
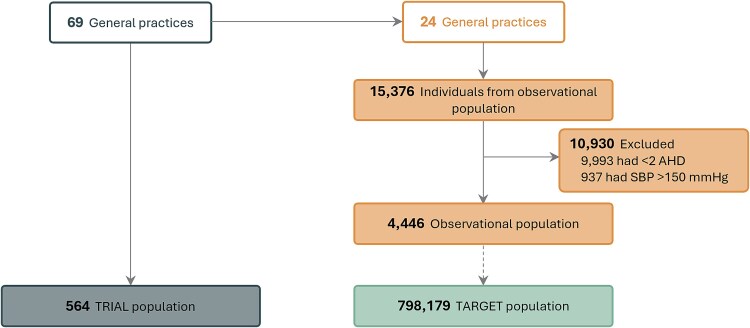
Study flowchart. AHD, antihypertensive drugs; SBP, systolic blood pressure.

We included 564/569 individuals (99.1%) from the OPTiMISE trial who were followed-up for an average of 4.1 years (Figure 2). From the 15 376 individuals available in the observational dataset, we selected 4446 individuals after applying the eligibility criteria (i.e. observational population). Based on these data, we estimated a target population of 798 179 individuals using the method described above (see ‘Statistical analyses’ section, Figure 1C).

### Baseline characteristics

Participants in the OPTIMISE trial had a median age of 84 years (IQR 82–87), similar to that of the target population (84 [81–87] years) (Table 1). However, there were fewer women in the trial (48.4%) compared with the target population (58.4%). The distribution of frailty differed between populations: only 11.3% of trial participants were classified as living with frailty compared with 25.5% in the target population, whereas 40.8% were considered fit in the trial versus only 14.9% in the target population. Cardiovascular polypharmacy was also more frequent in the target population (77.4% vs. 66.7%). Similarly, the prevalence of chronic conditions was consistently higher in the target population, including chronic kidney disease (38.2% vs. 32.8%), type 2 diabetes (23.9% vs. 17.9%), previous myocardial infarction (12.3% vs. 7.1%) and stroke or transient ischaemic attack (15.8% vs. 14.4%). Similar differences were observed on directly comparing the trial and observational populations (Appendix S1).

**Table 1:** Comparison of baseline characteristics of trial and target populations.

Baseline variables	OPTiMISE trial population *n* = 564; Median [P25; P75] or *n* (%)	Target population *n* = 798 179; Median [P25; P75] or *n* (%)	*P*-value
Age, in years	84 [82; 87]	84 [81; 87]	.215
Female (%)	273 (48.4)	465 977 (58.4)	<.001
Systolic blood pressure, in mmHg	131 [121; 141]	128.5 [124; 140]	.126
Diastolic blood pressure, in mmHg	69 [63; 75]	73.9 [63; 76.3]	<.001
BMI, in kg/m^2^	27.3 [24.6; 30.1]	25.9 [24.9; 31.2]	.117
Frailty score	-	-	<.001
Fit	230 (40.8)	119 129 (14.9)	-
Less fit	270 (47.9)	475 874 (59.6)	-
Frail	64 (11.3)	203 176 (25.5)	-
Polypharmacy (≥3 cardiovascular medications by day)	376 (66.7)	617 589 (77.4)	<.001
Chronic kidney disease	185 (32.8)	305 053 (38.2)	<.001
Type 2 diabetes	101 (17.9)	190 999 (23.9)	<.001
Myocardial infraction	40 (7.1)	98 039 (12.3)	<.001
Stroke/transient ischaemic attack	81 (14.4)	125 948 (15.8)	<.001

Comparisons between populations were assessed using Wilcoxon rank-sum test for continuous variables, and chi-square test for categorical variables. BMI, body mass index.

### Distribution of probabilities and IPW

The model estimating trial inclusion probabilities is shown in Appendix S2 of Appendices. Higher frailty levels and comorbidities were associated with lower estimated probabilities of trial participation, whereas higher SBP showed a modest positive association. However, as illustrated in Appendix S3 of Appendices, the absolute values of the predicted probabilities are uniformly low and show substantial overlap between trial and target populations. The low probabilities in both reflect the large difference in sample size between the real-world population and the trial population. Descriptive statistics for inclusion probabilities and stabilised IPWs are provided in Appendix S4 of Appendices; median inclusion probability was low (0.002), and stabilised IPWs showed moderate variability (median = 0.71, range: 0.05–6.19), supporting the robustness of the weighting process used for calibration.

### Calibrated effect estimates

**Figure 3:**
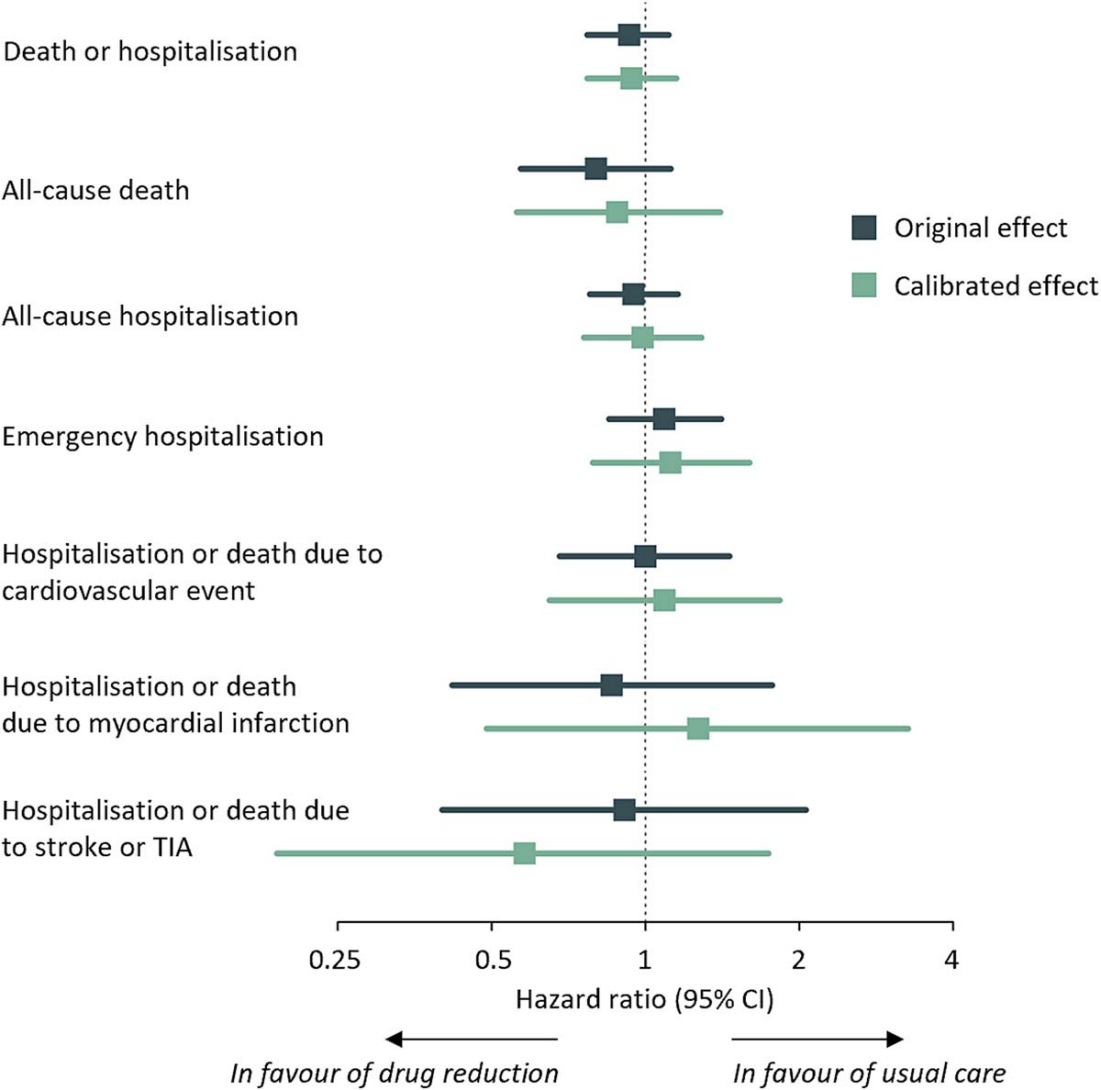
Original and calibrated effect of antihypertensive medication reduction on clinical outcomes in the OPTiMISE trial population.

After calibration, in the trial population, there was no evidence of a difference in all-cause hospitalisation or mortality at 4 years between the medication reduction and usual care groups [adjusted HR = 0.94 (95% CI: 0.77–1.15; *P* = .568)] (Table 2). This was similar to the non-calibrated risk of primary outcome reported in the original trial [adjusted HR = 0.93 (95% CI: 0.77–1.11)], despite a marginally wider confidence interval (Figure 3).

**Table 2:** Time-to-event analyses of clinical outcomes at follow-up in the OPTiMISE trial population after calibration to the target population.

Outcomes	Medication reduction group^a^ (*n* = 263; weighted *n* = 243)	Usual care group^a^ (*n* = 271; weighted *n* = 291)	Adjusted HR (95% CI)	*P*-value
All-cause hospitalisation or mortality (combined; primary outcome)	191.8 (79)	238.2 (81.8)	0.94 (0.77–1.15)	.568
All-cause mortality	68.6 (28.3)	92.5 (31.8)	0.88 (0.56–1.40)	.600
All-cause hospitalisation	182 (74.9)	233.6 (80.2)	0.99 (0.76–1.29)	.926
Emergency hospitalisation	118.8 (48.9)	129.7 (44.5)	1.12 (0.79–1.60)	.522
Hospitalisation or death due to major cardiovascular events	54.7 (22.5)	60.9 (20.9)	1.09 (0.65–1.83)	.749
Hospitalisation or death due to myocardial infarction	11 (4.5)	10.2 (3.5)	1.27 (0.49–3.26)	.624
Hospitalisation or death due to stroke	6.5 (2.7)	13.7 (4.7)	0.58 (0.19–1.74)	.328

^a^Number of participants with events (%)*.* Associations between the intervention and each time-to-event outcome were estimated using Cox proportional hazards models adjusted for baseline systolic blood pressure, with robust standard errors clustered at the trial-site level (and incorporating IPWs in calibrated analyses).

HR, Hazard ratio; 95% CI, 95% confidence interval.

For secondary outcomes, there was no evidence of a difference between groups after calibration: all-cause mortality (adjusted HR 0.88, 95% CI: 0.56–1.40), all-cause hospitalisation (0.99, 0.76–1.29), emergency hospitalisations (1.12, 0.79–1.60), major cardiovascular events (1.09, 0.65–1.83), myocardial infarction (1.27, 0.49–3.26) and stroke (0.58, 0.19–1.74) (Table 2). These results were consistent with the original non-calibrated trial estimates (e.g. adjusted HRs 0.80 for all-cause mortality, 0.95 for all-cause hospitalisation, 1.09 for emergency hospitalisations and 1.00 for major cardiovascular events), with calibration primarily resulting in slightly wider confidence intervals (Figure 3).

### Sensitivity analyses

In the sensitivity analysis excluding frailty from the inclusion models, while several coefficient estimates changed in magnitude, the direction and relative importance of most predictors were preserved (Appendix S5). The distribution of inclusion probabilities in the trial and target populations showed similar separation patterns, and the stabilised IPWs exhibited comparable ranges, with slightly reduced extreme values (maximum weight 3.27 vs. 6.19 in the main analysis) (Appendices S6 and S7).

## Discussion

We calibrated the OPTiMISE trial of antihypertensive deprescribing in older people to a representative population of older adults treated in routine primary care. After re-weighting trial participants to reflect the characteristics of the general primary care population in England, we obtained similar estimates to the original OPTiMISE report, suggesting that the trial findings are likely to be applicable to real-world populations. As expected, the calibration process widened the confidence intervals—an inherent consequence of reweighting except where the target population exactly matches the trial population [12, 21].

Prior work has shown that major hypertension trials frequently underrepresent older adults living with frailty and multiple long-term conditions—precisely those most vulnerable to the potential harms of intensive antihypertensive therapy [11, 22]. We confirmed this issue in our study: frailty was more than twice as prevalent in the real-world population compared with the trial, and chronic conditions were consistently more common. This selection pattern, a well-recognised source of bias in clinical trials, directly threatens external validity [10, 23]. Importantly, this challenge is not unique to our question; it is potentially highly relevant to many other causal clinical questions and to numerous trials that aim to represent older adults [24]. Although recent advances in causal inference methods that help to mitigate selection biases (e.g. target trial emulation), calibration offers an additional advantage by preserving the true randomisation of the original clinical trial and thus avoiding the confounding inherent to analyses based on real-world data [16]. Our calibrated efficacy estimates therefore reduces the uncertainty in applying the OPTiMISE trial results to real-world populations.

To our knowledge, there are no previous examples of trial calibration applied to antihypertensive deprescribing, nor more broadly to deprescribing interventions in other therapeutic areas. Nevertheless, trial calibration has been used in hypertension management and other cardiovascular conditions to generalise trial findings to more representative populations [25]. For example, Berkowitz and colleagues used calibration to transport the results of intensive blood pressure lowering trials to the US population, showing that while relative treatment effects were broadly preserved, absolute benefits and harms varied substantially in older and more comorbid individuals [26]. Similarly, Inoue and colleagues demonstrated that calibrating intensive antihypertensive treatment effects to populations with different ethnic distributions revealed meaningful heterogeneity in cardiovascular benefits and risks [27]. Finally, calibration approaches used by Wei and colleagues in heart failure populations have shown that treatment effects observed in selected trial participants can be meaningfully generalised to older, multimorbid real-world populations, albeit with attenuated absolute benefits [14]. Together, these studies support the relevance of calibration methods in cardiovascular medicine and provide a useful methodological comparison for our application of trial calibration to antihypertensive deprescribing.

This study also provides a methodological contribution relevant to future calibration research. Because the observational dataset only partially overlapped with the original OPTiMISE trial centres, direct calibration using the raw observational population would have violated key assumptions [17]. We therefore reconstructed a ‘virtual’ target population, scaling clinical characteristics observed in primary care to national age-sex strata. This approach enabled us to consider the trial population as being fully nested within the target population, satisfying identifiability assumptions and preventing bias resulting from sample overlap. To our knowledge, this is a novel application of target population reconstruction within a calibration framework and demonstrates its feasibility in contexts, where datasets are not fully independent. This strategy may be of particular relevance in other clinical domains facing similar structural constraints.

These findings have several important implications for clinical practice. First, they suggest that the OPTiMISE trial results can be generalised to the broader population of adults aged 80 years and older treated in primary care, including those living with frailty and multiple long-term conditions who were underrepresented in the original trial. Then, our results support emerging evidence from other deprescribing trials conducted in older populations. In particular, the RETREAT FRAIL trial similarly found no evidence that antihypertensive deprescribing was associated with increased mortality among older adults living with frailty in nursing homes, reinforcing the safety of deprescribing in this population [28]. Finally, for general practitioners and other clinicians, when discussing the risks and benefits of antihypertensive treatment with older patients, our results provide evidence that stopping one antihypertensive medication is unlikely to result in significant harm. These findings therefore mainly support the safety of deprescribing when it is undertaken in routine practice, rather than indicating that antihypertensive treatment should be systematically reduced. Importantly, they also indicate that, at present, there is no strong evidence that deprescribing antihypertensive treatment confers substantial clinical benefit.

Strengths of the study include the fact that the analysis does not break randomisation (therefore avoids introducing confounding) and detailed information on both the trial and target populations, including on key characteristics of concern such as polypharmacy and frailty. However, the study has several limitations. First, some variables were not available for the re-weighting such as practice-level clustering variables, cognitive and functional status. However, our calibrated estimates were robust to different specifications of the trial inclusion model, and it seems unlikely that adding these variables to the trial inclusion model would have considerably affected the results. Second, some variables in the target population could not be matched perfectly to the trial data due to the retrospective nature of routine data collection (e.g. frailty). A substantial effort was made to harmonise both datasets to minimise this issue. Third, our analyses relied on the virtual reconstruction of the target population, which assumes that—conditional on age and sex—the observational population is broadly representative of older adults treated in England. The final calibrated effect estimates are unlikely to be sensitive to the correctness of this assumption, given that the effect estimates on calibrating to the target and observational populations were so similar. The observational dataset used to inform the reconstruction of the target population was derived from a limited number of general practices that volunteered to participate in the trial and recruit participants, and may therefore not fully capture the diversity of primary care settings across England; although the populations served by these practices were varied, as reflected in the characteristics of registered patients, the representativeness of this sample cannot be formally verified, and some degree of selection bias cannot be excluded. Overall, these results support the generalisability of OPTiMISE findings to real-world older adults and suggest that calibration may be used more widely in analysing trials in older people.

## Supplementary Material

Supplementary_materials_afag192

## Data Availability

Requests for sharing of de-identified individual participant data and a data dictionary defining each field in the set will be considered by the corresponding author. The study protocol is available in the supplementary appendix and on our trial website (https://www.phctrials.ox.ac.uk/studies/optimise). All R codes and additional data (e.g. fitted models) are openly shared on GitHub [20]
