# Supplementary material for: Effect of antihypertensive medication reduction on short-term blood pressure control in older adults: calibration of OPTiMISE trial results to real-world primary care data
Source: Age Ageing. 2026 Jul 1;55(6):afag192. doi: 10.1093/ageing/afag192 (PMC13318848; doi:10.1093/ageing/afag192)
Supplement: Supplementary_materials_afag192 [file supplementary_materials_afag192.docx]

APPENDICES

**Effect of Antihypertensive medication reduction on short-term blood pressure control in older adults: Calibration of OPTiMISE trial results to real-world primary care data**

TABLE OF CONTENTS

Appendix 1. Comparison of baseline characteristics of trial and observation population (before reconstruction of the target population)

Appendix 2. Model of inclusion probability on the target population

Appendix 3. Distribution of inclusion probabilities in the trial and target populations.

**Appendix 4.** Descriptive statistics of rescaled normalised probabilities of inclusion, and of stabilized normalized IPW in the trial population (n=564)

**Appendix 5.** Model of inclusion probability on the estimated true target population, without taking into account frailty (sensitivity analysis)

**Appendix 6.** Distribution of inclusion probabilities in the trial and target populations, without taking into account frailty (sensitivity analysis)

**Appendix 7**. Descriptive statistics of rescaled normalised probabilities of inclusion, and of stabilized normalized IPW in the trial population (n=564), without taking into account frailty (sensitivity analysis)

**Appendix 1.** Comparison of baseline characteristics of trial and observation population (before reconstruction of the target population)

| **Baseline variables** | **OPTiMISE trial population**  *n*=564 | **Observational population**  *n*=4,446 | ***p-value^*^*** |
| --- | --- | --- | --- |
| Age, *in years* | 84 [82 ; 87] | 84 [82 ; 87] | 0.742 |
| Female (*%*) | 273 (48.4) | 2,556 (57.5) | <0.001 |
| Systolic blood pressure, *in mmHg* | 131 [121 ; 141] | 130 [122 ; 140] | 0.339 |
| Diastolic blood pressure, *in mmHg* | 69 [63 ; 75] | 70 [64 ; 78] | <0.001 |
| BMI, *in kg/m^2^* | 27.3 [24.6 ; 30.1] | 26.8 [24 ; 30.3] | 0.172 |
| Frailty score | - | - | <0.001 |
| Fit | 230 (40.8) | 661 (14.9) | - |
| Less fit | 270 (47.9) | 2644 (59.5) | - |
| Frail | 64 (11.3) | 1141 (26.7) | - |
| Polypharmacy (≥3 cardiovascular medications by day) | 376 (66.7) | 3,437 (77.3) | <0.001 |
| Chronic kidney disease | 185 (32.8) | 1,707 (38.4) | <0.001 |
| Type 2 diabetes | 101 (17.9) | 1,063 (23.9) | <0.001 |
| Myocardial infraction | 40 (7.1) | 553 (12.4) | <0.001 |
| Stroke/Transient ischaemic attack | 81 (14.4) | 707 (15.9) | <0.001 |

*Legend*: Comparisons between populations were assessed using Wilcoxon rank-sum test for continuous variables, and chi-square test for categorical variables. *Abbreviations*: BMI, Body mass index.

**Appendix 2.** Model of inclusion probability on the target population


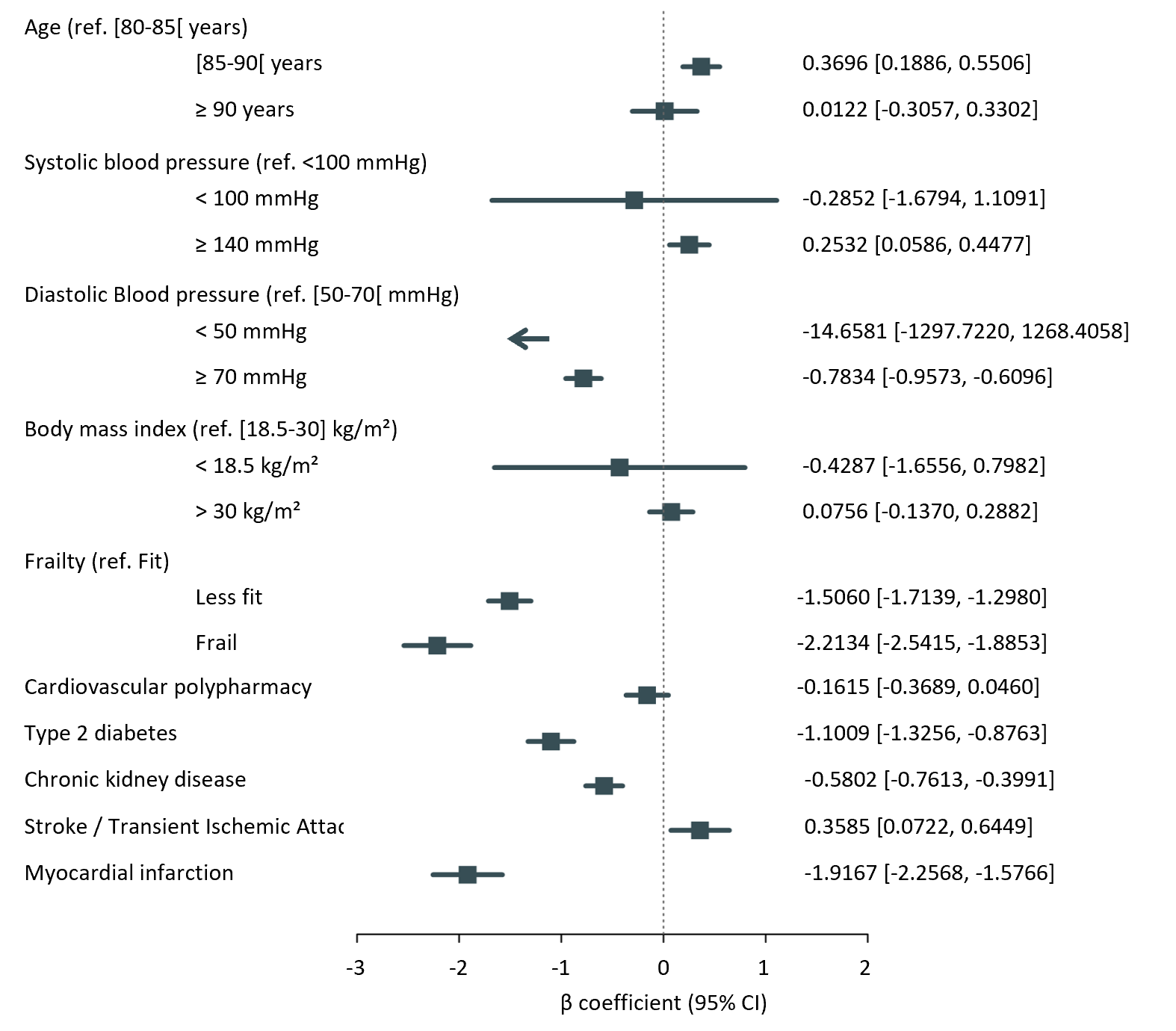


***Legend*: The results presented in this table are β coefficients obtained from regression models estimated across multiple imputed datasets. Final estimates were pooled using Rubin’s rules to account for the uncertainty introduced by missing data.**

**Appendix 3.** Distribution of inclusion probabilities in the trial and target populations.


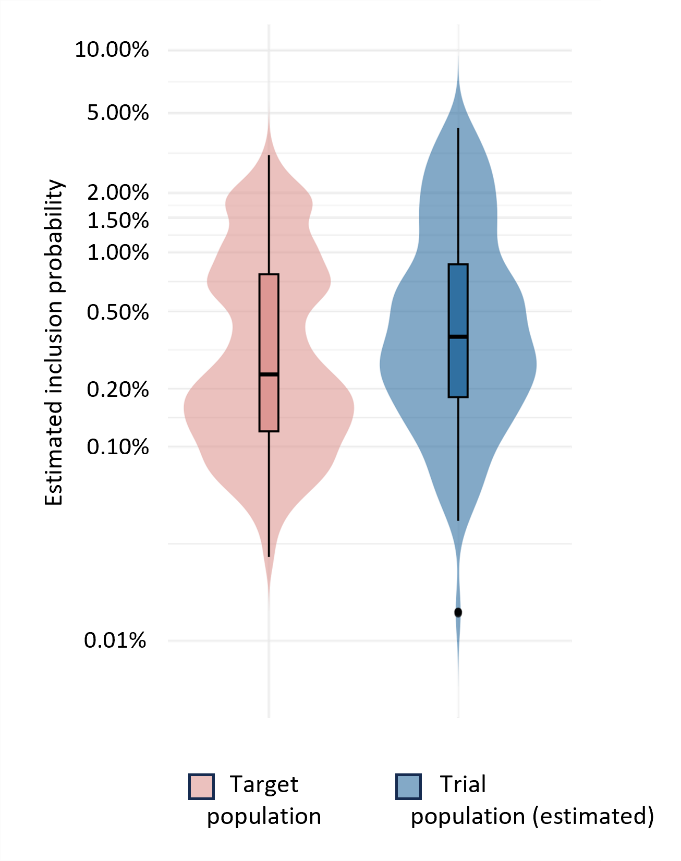


***Legend*:** Violin and box plots showing the distribution of predicted probabilities of inclusion in the trial (i.e., probability of belonging to the trial sample vs the target population), estimated from a logistic model fitted on the merged dataset. Distributions are presented separately for individuals from the target and trial populations. This figure allows assessment of the degree of overlap (positivity) between the two populations.

**Appendix 4.** Descriptive statistics of rescaled normalised probabilities of inclusion, and of stabilized normalized IPW in the trial population (n=564)

| **Metrics** | **Min IPW** | **Q1** | **Median** | **Mean** | **Q3** | **Max IPW** |
| --- | --- | --- | --- | --- | --- | --- |
| Rescaled probability of inclusion | 0.000271 | 0.001203 | 0.002361 | 0.00537 | 0.007714 | 0.030998 |
| Stabilized IPW (normalized) | 0.05364 | 0.216987 | 0.709935 | 1.0 | 1.384555 | 6.18927 |

*Abbreviations*: IPW, Inverse probability weight.

**Appendix 5.** Model of inclusion probability on the estimated true target population, without taking into account frailty (sensitivity analysis)

| **Variable** | **Pooled estimate of the β coefficient** | **SE** | **Lower** | **Upper** |
| --- | --- | --- | --- | --- |
| (Intercept) | -6.9065 | 0.112 | -7.1259 | -6.687 |
| Age, [85,90[ years | 0.2969 | 0.0924 | 0.1157 | 0.478 |
| Age, ≥90 years | -0.2341 | 0.1617 | -0.5509 | 0.0827 |
| SBP, <100 mmHg | -0.3473 | 0.711 | -1.7409 | 1.0462 |
| SBP, ≥140 mmHg | 0.2906 | 0.0992 | 0.0962 | 0.485 |
| DBP, <50 mmHg | -13.5375 | 432.2982 | -860.8263 | 833.7514 |
| DBP, ≥70 mmHg | -0.6808 | 0.0882 | -0.8537 | -0.508 |
| BMI, <18.5 *kg/m^2^* | -0.5624 | 0.6167 | -1.7838 | 0.6589 |
| BMI, >30 *kg/m^2^* | -0.0077 | 0.1082 | -0.2207 | 0.2054 |
| Frailty, less fit | -0.819 | 0.0955 | -1.0062 | -0.6318 |
| Frailty, frail | -1.2826 | 0.1129 | -1.504 | -1.0612 |
| Polypharmacy (≥ 3 cardiovascular medications by day) | -0.6727 | 0.0919 | -0.8529 | -0.4925 |
| Type 2 diabetes | -0.1279 | 0.1376 | -0.3976 | 0.1418 |
| Chronic kidney disease | -2.0517 | 0.1728 | -2.3903 | -1.713 |
| Stroke/Transient ischaemic attack | -6.9065 | 0.112 | -7.1259 | -6.687 |
| Myocardial infraction | 0.2969 | 0.0924 | 0.1157 | 0.478 |

***Legend*: The results presented in this table are β coefficients obtained from regression models estimated across multiple imputed datasets. Final estimates were pooled using Rubin’s rules to account for the uncertainty introduced by missing data. *Abbreviations:* SE, Standart error.**

**Appendix 6.** Distribution of inclusion probabilities in the trial and target populations, without taking into account frailty (sensitivity analysis)


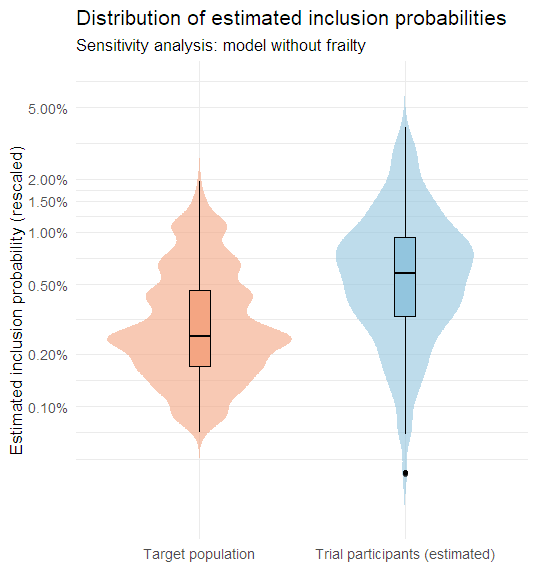


***Legend*:** Violin and box plots showing the distribution of predicted probabilities of inclusion in the trial (i.e., probability of belonging to the trial sample vs the target population), estimated from a logistic model fitted on the merged dataset excluding frailty. Distributions are presented separately for individuals from the target and trial populations. The y-axis is displayed on a logarithmic scale. This sensitivity analysis assesses the impact of excluding frailty on the degree of overlap (positivity) between populations.

**Appendix 7.** Descriptive statistics of rescaled normalised probabilities of inclusion, and of stabilized normalized IPW in the trial population (n=564), without taking into account frailty (sensitivity analysis)

| **Metrics** | **Min IPW** | **Q1** | **Median** | **Mean** | **Q3** | **Max IPW** |
| --- | --- | --- | --- | --- | --- | --- |
| Rescaled probability of inclusion | 0.000709 | 0.001705 | 0.002527 | 0.00383 | 0.004637 | 0.019291 |
| Stabilized IPW (normalized) | 0.119631 | 0.498214 | 0.914224 | 1.0 | 1.356409 | 3.274821 |

*Abbreviations*: IPW, Inverse probability weight.
